# Supplementary material for: DMRforPairs: identifying Differentially Methylated Regions between unique samples using array based methylation profiles
Source: BMC Bioinformatics. 2014 May 15;15:141. doi: 10.1186/1471-2105-15-141 (PMC4046028; doi:10.1186/1471-2105-15-141)
Supplement: Additional file 2 — DMRforPairs output for the comparison of A431-MCF7 and NA17018-NA17105. Please start from the HTML files in each folder. Available via the BMC Bioinformatics website. [file 1471-2105-15-141-S2.zip › 1394847754114233_MOESM2_ESM/A431_MCF7/figures/298.pdf]

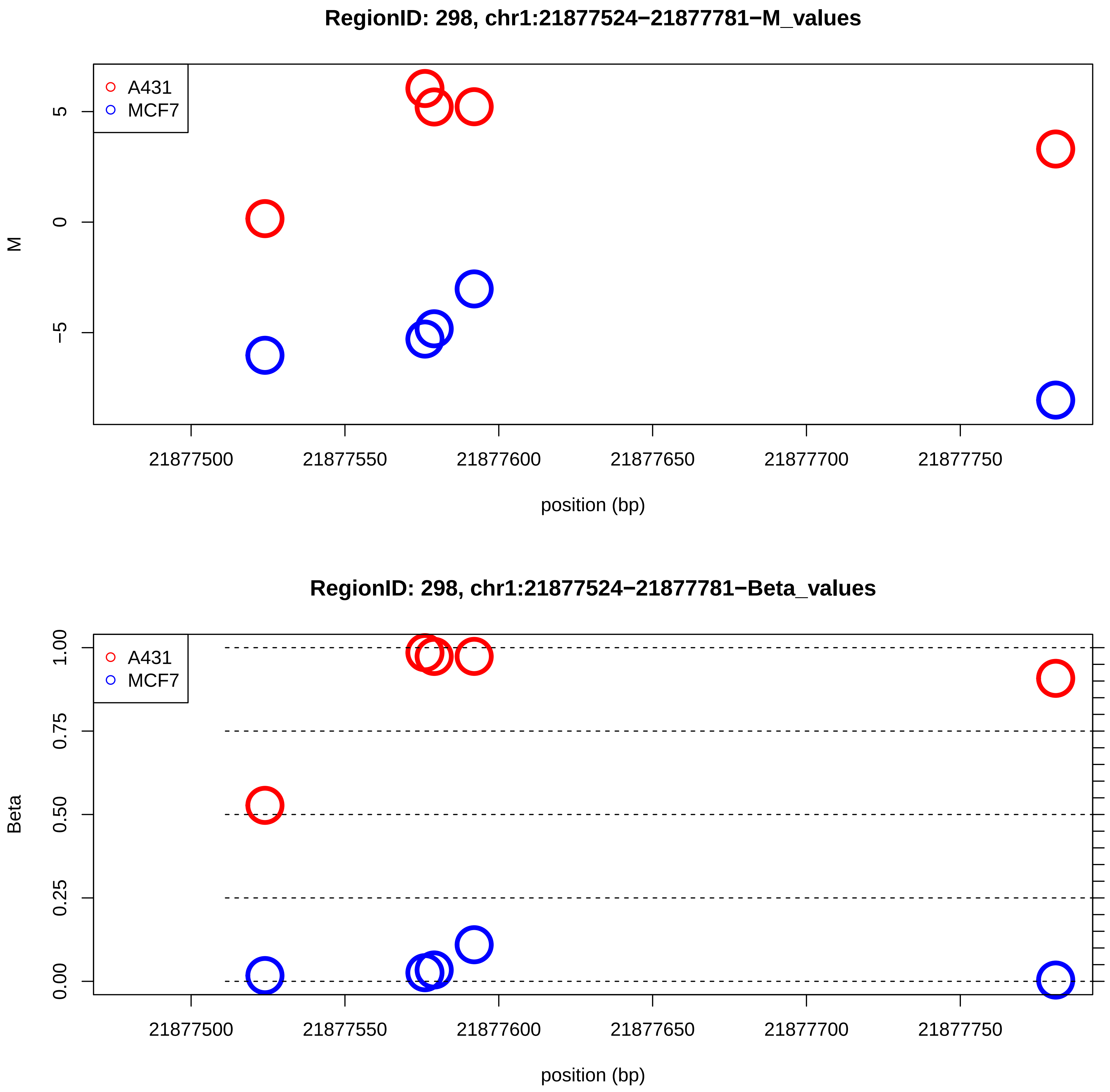
RegionID: 298, chr1:21877524−21877781−M_values
A431
MCF7
M
5
0
−5
21877500
21877550
21877600
21877650
21877700
21877750
position (bp)
RegionID: 298, chr1:21877524−21877781−Beta_values
A431
MCF7
Beta
1.00
0.75
0.50
0.25
0.00
21877500
21877550
21877600
21877650
21877700
21877750
position (bp)

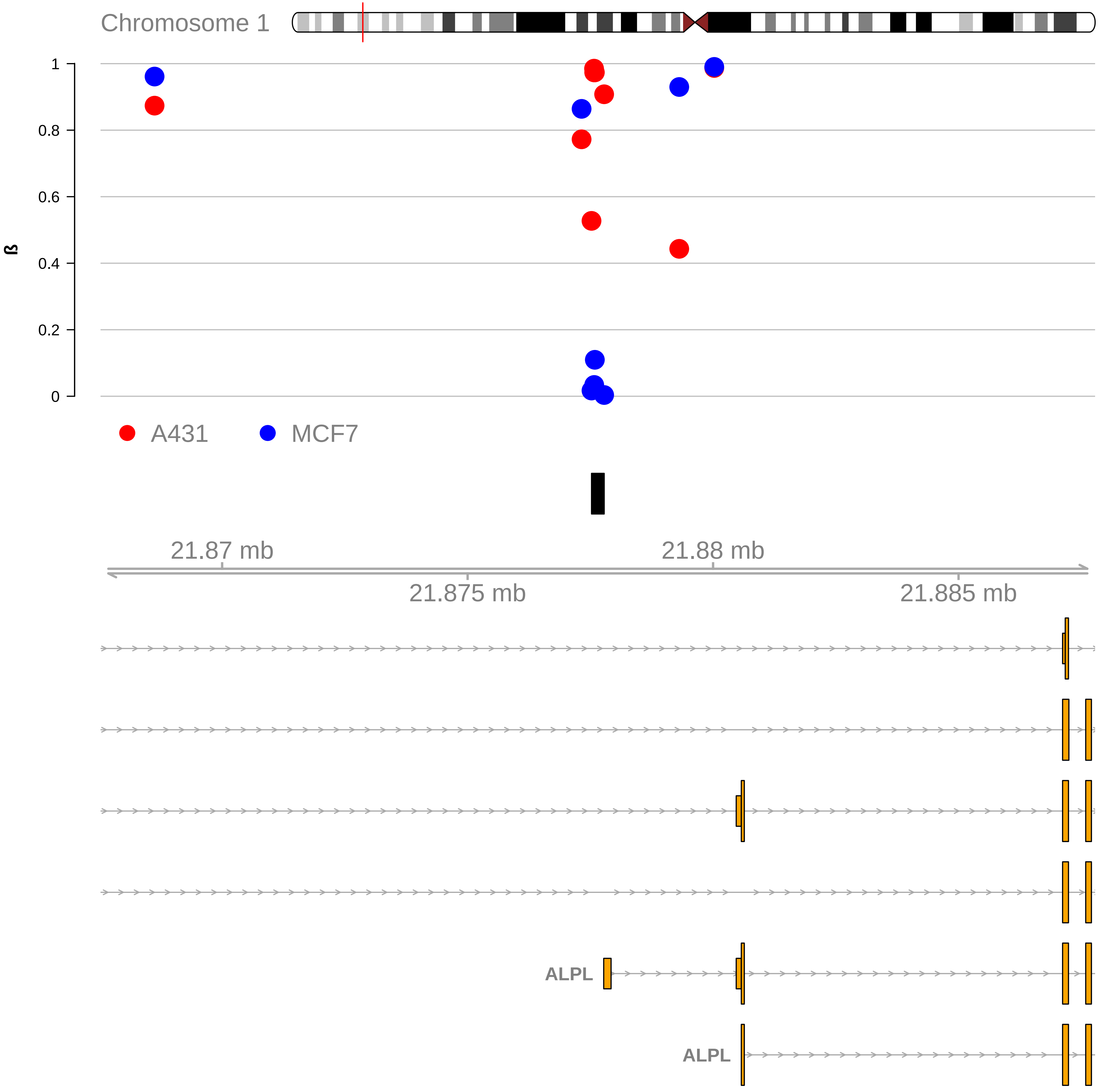

Chromosome 1
1
0.8
0.6
0.4
0.2
0
ß
A431
MCF7
21.87 mb
21.88 mb
21.875 mb
21.885 mb
ALPL
ALPL
